# RNA-seq analysis identifies cytoskeletal structural genes and pathways for meat quality in beef

**DOI:** 10.1371/journal.pone.0240895

**Published:** 2020-11-11

**Authors:** Joel D. Leal-Gutiérrez, Mauricio A. Elzo, Chad Carr, Raluca G. Mateescu

**Affiliations:** Department of Animal Sciences, University of Florida, Gainesville, Florida, United States of America; Universidade Federal de Viçosa, BRAZIL

## Abstract

RNA sequencing (RNA-seq) has allowed for transcriptional profiling of biological systems through the identification of differentially expressed (DE) genes and pathways. A total of 80 steers with extreme phenotypes were selected from the University of Florida multibreed Angus-Brahman herd. The average slaughter age was 12.91±8.69 months. Tenderness, juiciness and connective tissue assessed by sensory panel, along with marbling, Warner-Bratzler Shear Force (WBSF) and cooking loss, were measured in *longissimus dorsi* muscle. Total RNA was extracted from muscle and one RNA-seq library per sample was constructed, multiplexed, and sequenced based on protocols by Illumina HiSeq-3000 platform to generate 2×101 bp paired-end reads. The overall read mapping rate using the Btau_4.6.1 reference genome was 63%. A total of 8,799 genes were analyzed using two different methodologies, an expression association and a DE analysis. A gene and exon expression association analysis was carried out using a meat quality index on all 80 samples as a continuous response variable. The expression of 208 genes and 3,280 exons from 1,565 genes was associated with the meat quality index (p-value ≤ 0.05). A gene and isoform DE evaluation was performed analyzing two groups with extreme WBSF, tenderness and marbling. A total of 676 (adjusted p-value≤0.05), 70 (adjusted p-value≤0.1) and 198 (adjusted p-value≤0.1) genes were DE for WBSF, tenderness and marbling, respectively. A total of 106 isoforms from 98 genes for WBSF, 13 isoforms from 13 genes for tenderness and 43 isoforms from 42 genes for marbling (FDR≤0.1) were DE. Cytoskeletal and transmembrane anchoring genes and pathways were identified in the expression association, DE and the gene enrichment analyses; these proteins can have a direct effect on meat quality. Cytoskeletal proteins and transmembrane anchoring molecules can influence meat quality by allowing cytoskeletal interaction with myocyte and organelle membranes, contributing to cytoskeletal structure and architecture maintenance postmortem.

## Introduction

Meat quality phenotypes in beef cattle are economically important traits which are quantitative in nature with usually low to medium genetic control [1,2]. Multiple efforts have been directed to identify genes able to explain part of the phenotypic variability present in meat quality related traits in different populations [3–5]. Large-scale genotyping platforms, high-density panels of molecular markers, and genome-wide association (GWA) analyses are extensively used to identify major genes for improvement of meat quality traits in beef cattle [6–8]. However, our knowledge about the exact mechanism through which the identified genomic regions contribute to phenotypic variability in quantitative traits is still very limited. This could be partially due to alterations at transcriptional level and splicing events [9,10] which are not captured at the DNA level.

Recently, RNA-seq has allowed for transcriptional profiling of biological systems through the identification of differentially expressed (DE) genes and pathways in order to identify biological mechanisms associated with the phenotypic condition being assessed [11]. Understanding the biological mechanisms associated with complex and economically important traits would help identify genes that could potentially be used as biomarkers in animal selection [12]. Differential expression is most often derived from comparing two or more conditions; however, converting a continuous phenotype such as meat quality into categories leads to loss of phenotypic variability. Seo et al. [13] demonstrated that expression analysis based on robust regression, which performs an association between a continuous trait and mRNA expression, achieves a lower false discovery rate and higher precision. This approach is referred to as expression association analysis in the present document.

The objectives of the present research were to perform: 1) a gene and exon expression association analysis for a continuous meat quality index defined through a principal component analysis of meat quality related traits; and 2) a gene and isoform differential expression for WBSF, tenderness and marbling as categorical variables using a crossbred Brahman-Angus population in order to identify gene whose expression is able to explain phenotypic variability associated to meat quality.

## Materials and methods

### Cattle population and phenotypic data

The research protocol was approved by the University of Florida Institutional Animal Care and Use Committee (201003744). A total of 120 steers born between 2013 and 2014 were included in the analysis. The animals belong to the multibreed Angus-Brahman herd from the University of Florida [14–16]. Cattle were classified into three different groups based on their expected Angus and Brahman breed composition. Based on the Angus composition determined using pedigree information, the grouping was as follows: 1 = 100 to 65%; 2 = 64% to 40%; 3 = 39 to 0% [17].

Steers were transported to a commercial packing plant when their subcutaneous fat thickness over the ribeye reached 1.27 cm. The average slaughter weight was 573.34±54.79 kg at 12.91±8.69 months. The steers were harvested using established USDA-FSIS procedures, and 5–10 g of the *longissimus dorsi* muscle was sampled after splitting the carcass. The sample was snapped-frozen in liquid nitrogen and stored at -80°C for RNA extraction. Marbling was recorded 48 hours postmortem in the ribeye muscle at the 12th/13th rib interface by visual appraisal. The following numerical scale was used for marbling: Practically Devoid = 100–199, Traces = 200–299, Slight = 300–399, Small = 400–499, Modest = 500–599, Moderate = 600–699, Slightly Abundant = 700–799, Moderately Abundant = 800–899, Abundant = 900–999.

Two 2.54 cm steaks from the *longissimus dorsi* muscle at the 12th/13th rib interface were sampled from each animal. The first steak was used to measure WBSF and cooking loss, and the second steak was used to measure tenderness, juiciness and connective tissue by a sensory panel. The steaks were transported to the Meat Science Laboratory of the University of Florida, aged for 14 days at 1 to 4°C, and then stored at −20°C. Both frozen steaks from each animal were allowed to thaw at 4°C for 24 hours and cooked to an internal temperature of 71°C on an open-hearth grill. After cooking, the first steak was cooled at 4°C for 18 to 24 hours and used to measure WBSF and cooking loss according to the American Meat Science Association Sensory Guidelines [18]. Six cores with a 1.27-cm diameter and parallel to the muscle fiber were sheared with a Warner-Bratzler head attached to an Instron Universal Testing Machine (model 3343; Instron Corporation, Canton, MA). The Warner-Bratzler head moved at a cross head speed of 200 mm/min. The average peak load (kg) of six cores from the same animal was calculated. The weight lost during cooking was recorded and cooking loss was expressed as a percentage of the cooked weight out of the thaw weight.

Tenderness, juiciness and connective tissue were measured by a sensory panel following the American Meat Science Association Sensory Guidelines [18]. The sensory panel consisted of eight to eleven trained members, and steaks from six animals were assessed per session. Two 1 × 2.54 cm samples from each steak were provided to each panelist. Sensory panel measurements analyzed by the sensory panelists included: tenderness (8 = extremely tender, 7 = very tender, 6 = moderately tender, 5 = slightly tender, 4 = slightly tough, 3 = moderately tough, 2 = very tough, 1 = extremely tough), juiciness (8 = extremely juicy, 7 = very juicy, 6 = moderately juicy, 5 = slightly juicy, 4 = slightly dry, 3 = moderately dry, 2 = very dry, 1 = extremely dry), and connective tissue (8 = none detected, 7 = practically none, 6 = traces amount, 5 = slight amount, 4 = moderate amount, 3 = slightly abundant, 2 = moderately abundant, 1 = abundant amount). For each phenotype, the average score by steak from all members of the panel was analyzed.

### Sample selection for RNA sequencing

A principal component analysis using marbling, WBSF, cooking loss, juiciness, tenderness and connective tissue was performed on 120 steers using PROC FACTOR procedure of SAS [19], and the first three principal components (PC) were used to construct a meat quality index for each animal as a pseudo-phenotype. The meat quality index was calculated using the following formula:

$${Meat\;quality\;index}_{i}=\sum_{j=1}^{3}\left({PCS}_{ij}*{PCW}_{j}\right)$$ (1)

Where PCS_ij_ is the score of the animal i for the PC_j_, and PCW_j_ is the weight of the PC_j_ represented by the amount of variability explained by each PC (eigenvalues). The amount of variance explained by PC_1_, PC_2_ and PC_3_ was 44.26%, 20.04% and 13.29%, respectively. Given that the summation of principal component scores for each PC was zero, the minimum value of each PC was added as a constant in order to have only positive PCS values. The relationship between the meat quality index and WBSF, cooking loss, juiciness, tenderness, and connective tissue is presented in S1 Fig. Higher meat quality index was associated with higher marbling, and more tender and juicy meat.

The meat quality index was used to rank the animals from low to high performance. Out of the 120 steers, 80 animals with extreme low and high meat quality index were selected and used for RNA sequencing [20].

### RNA-seq library preparation and sequencing

Total RNA was extracted from muscle using TRIzol® reagent (Thermo Fisher Scientific, Waltham, MA, USA) according to the manufacturer’s protocol (Invitrogen, catalog no. 15596–026). RNA concentration was measured using a NanoDrop 2000 spectrophotometer (Thermo Fisher Scientific, Waltham, MA, USA) and RNA integrity was verified by formaldehyde gel. Total RNA samples were sent to RAPiD Genomics LLC (Gainesville, Florida, United States) for mRNA isolation, RNA-seq library preparation and sequencing procedures after verifying RNA quality by using the RIM parameter. One RNA-seq library for each sample was constructed, multiplexed, and sequenced based on protocols of Illumina HiSeq 3000 PE100 platform (Illumina, San Diego, CA, USA). All samples were sequenced on 8 lanes, generating 2×101 nts paired-end reads. RNA-seq data is available at the European Nucleotide Archive, accession number PRJEB31379, https://www.ebi.ac.uk/ena/data/search?query=PRJEB31379.

### Read alignment and counting

The pipeline described by Korpelainen et al. [21] was used to generate an index for the Btau_4.6.1 reference genome, and to obtain the gene and exon counts and isoform FPKM (Fragments Per Kilobase of exon per Million fragments mapped) files. Tophat 2.1.0 [22], Bowtie2 2.3.4 [23], Picard [24] and samtools [25] were used to generate the Btau_4.6.1 index. Eight forward and eight reverse FASTQ files per sample were concatenated in separated FASTQ files and analyzed with FastQC 0.9.6 [26] to check quality of the raw sequence reads. Read trimming was performed with PRINSEQ 0.20.4 [27] using 3 bp sliding windows and a phred threshold of 20. Reads with more than 2 ambiguous bases were discarded. Cutadapt 1.8.1 [28] was used to remove adapter sequences keeping only reads with a minimum length of 50 nts.

Tophat 2.1.0 [22] and Bowtie2 2.3.4 [23] were used to perform paired-end read mapping against the Btau_4.6.1 reference genome [29]. RSeQC 2.6.4 [30] was employed for obtaining alignment statistics such as gene body coverage, junction annotation, junction saturation and paired-end read inner distance size. Paired-end read counts for all annotated genes were generated using HTSeq 0.9.1 [31] from paired-end reads uniquely mapped. Cufflinks 2.2.1.1 [10,32] was used to assemble transcripts and estimate transcript abundance in FPKM. The RNA-seq differential expression analysis pipeline DEXSeq [33,34] was used to determine exon counts per gene. Samtools 1.9 [25] was used for indexing and sorting of the alignment files several times through the pipeline. Genes and exons with less than 10 counts on average were excluded from the analysis.

### Gene and exon expression association analysis for meat quality index

The procedure described by Seo et al. [13] was utilized to perform the expression association analysis by gene and exon for the continuous meat quality index including all 80 sequenced samples. Gene and exon counts were normalized using trimmed mean of M-values (TMM) normalization method available in the R package edgeR [35–37]. The R packages sfsmisc and MASS [36,38,39] were used to compute the Huber’s M-estimator based robust regression. In the robust regression analysis, the meat quality index was the response variable, and normalized gene or exon counts, the first PC from the “PCA for population structure” work-flow of JMP [40] and year of birth of the animal were included as fixed effects. A total of 8,799 genes and 96,645 exons were tested in this analysis.

### Gene and isoform differential expression analysis

Out of the 80 samples selected for sequencing, sets of 40 animals with extreme values for WBSF, tenderness and marbling were used in the DE procedure. Within each set, samples were classified into two categories based on the phenotype: tender or tough (for WBSF and tenderness) and high or low marbling.

The methodology described by Korpelainen et al. [21] was used for the identification of DE genes which utilizes the R package DESeq2 1.20.0 [41]. Year of birth, breed group and a categorical classification based on phenotype were included as fixed effects in the analysis. A total of 8,799 genes were analyzed for differential gene expression. Genes with a Benjamini-Hochberg adjusted p-values lower than 0.05 for WBSF and 0.1 for tenderness and marbling were considered to be DE.

The DE isoform analysis was performed with MetaDiff [42]. Year of birth, breed group and the categorical classification based on phenotype were included as fixed effects in the model. Only genes with alternative splicing were analyzed, and isoforms with less than 10 FPKM across samples were excluded. A total of 957 genes with 4,471 isoforms were included in the DE isoform analysis, and a false discovery rate (FDR) threshold of 0.1 was used to identify DE isoforms.

### Gene enrichment analysis

The R packages GOglm and goseq [36,43,44] were used to identify enriched GO terms. Four gene lists resulting from the gene expression association analysis and from the DE gene analysis for WBSF, tenderness and marbling were assessed. GO terms with fewer than 30 annotated genes were excluded. The GO terms established as enriched had unadjusted p-values lower than 0.05.

### Protein modeling

Twelve genes were selected for further analysis. The protein sequences were obtained from ensembl [45]. Protein models were constructed using the SwissModel server [46–48], and visualized and edited using the software DeepView v4.1 [49]. TMHMM2.0 [50], PROSITE [51] and SignalIP 4.0 servers [52] were used for predicting transmembrane regions, protein domains and signal peptide localization, respectively.

### Protein-protein interaction network

Thirty genes identified across analysis were used as query in the IntAct database [53] including only human proteins. The protein-protein interaction network was visualized using Cytoscape 3.7.2 [54].

## Results

### Phenotypic data

Table 1 shows the phenotypic distribution of the meat quality phenotypes for the animals used in this study.

**Table 1:** Descriptive statistics for the meat quality phenotypes and the constructed meat quality index.

		Mean	SD	Maximum	Minimum	N
Meat quality index		2.34	0.57	3.35	1.15	80
WBSF (kgs)	Tender	2.84	0.23	3.20	2.30	20
	Tough	5.61	0.51	6.90	5.02	20
Tenderness	Tender	6.24	0.21	6.60	5.90	20
	Tough	4.00	0.50	4.50	3.00	20
Marbling	Low	321	19.17	360	300	20
	High	576	50.93	650	500	20

The phenotypes were recorded in *longissimus dorsi* muscle from a multibreed Angus-Brahman population.

### Paired-end read alignment and paired-end read counting

After excluding single reads, and fitering out bases and reads with low sequencing quality, on average, 31.9 million high-quality paired reads were included in the analysis and mapped using the Btau_4.6.1 reference genome. The overall read mapping rate was 63% and the mean fragment inner distance was 144.2±64.46 bases (S1 Table).

### Expression association analysis for the meat quality index

#### Gene expression association analysis

**Fig 1:**
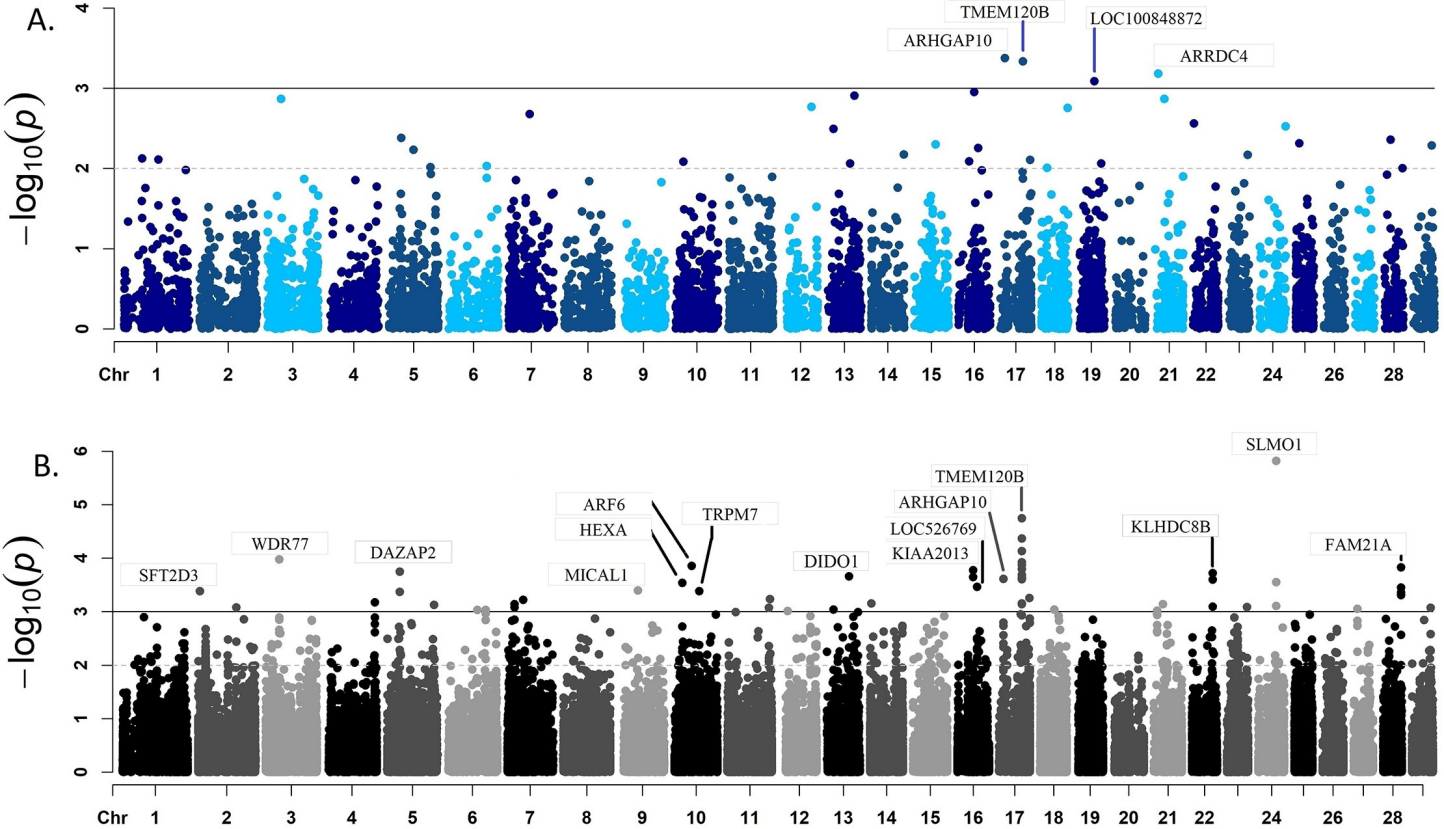
Results from the association analysis between gene expression (A) or exon expression (B) and meat quality index. The x-axis represents the location of the gene or exon across the bovine genome. The black line shows a p-value threshold of 1x10^-3^.

Expression of 208 genes was associated with the meat quality index (S2 Table p-value ≤ 0.05). The *Rho GTPase Activating Protein 10* (*ARHGAP10*), *Transmembrane Protein 120B* (*TMEM120B*), *Arrestin Domain Containing 4* (*ARRDC4*), *KIAA2013*, *NDRG Family Member 3* (*NDRG3*), *WD Repeat Domain 73* (*WDR73*) and *WD Repeat Domain 77* (*WDR77*) genes encode cytoskeletal associated proteins and were identified as highly associated (p-value ≤ 1x10^-4^) with the meat quality index (Fig 1A).

#### Exon expression association analysis

A total of 3,280 exons from 1,565 genes were associated with the meat quality index (p-value ≤ 0.05) (S2 Table and Fig 1B). The *SLMO1* (also named *PRELID3A*), *TMEM120B*, *WDR77*, *ADP Ribosylation Factor 6* (*ARF6*), *FAM21A*, *KIAA2013*, *DAZ Associated Protein 2* (*DAZAP2*), *Kelch Domain Containing 8B* (*KLHDC8B*), and *Death Inducer-Obliterator 1* (*DIDO1*) genes had at least one exon highly associated with meat quality index in the present analysis.

### Differential expression analysis

#### Differentially expressed genes

**Fig 2:**
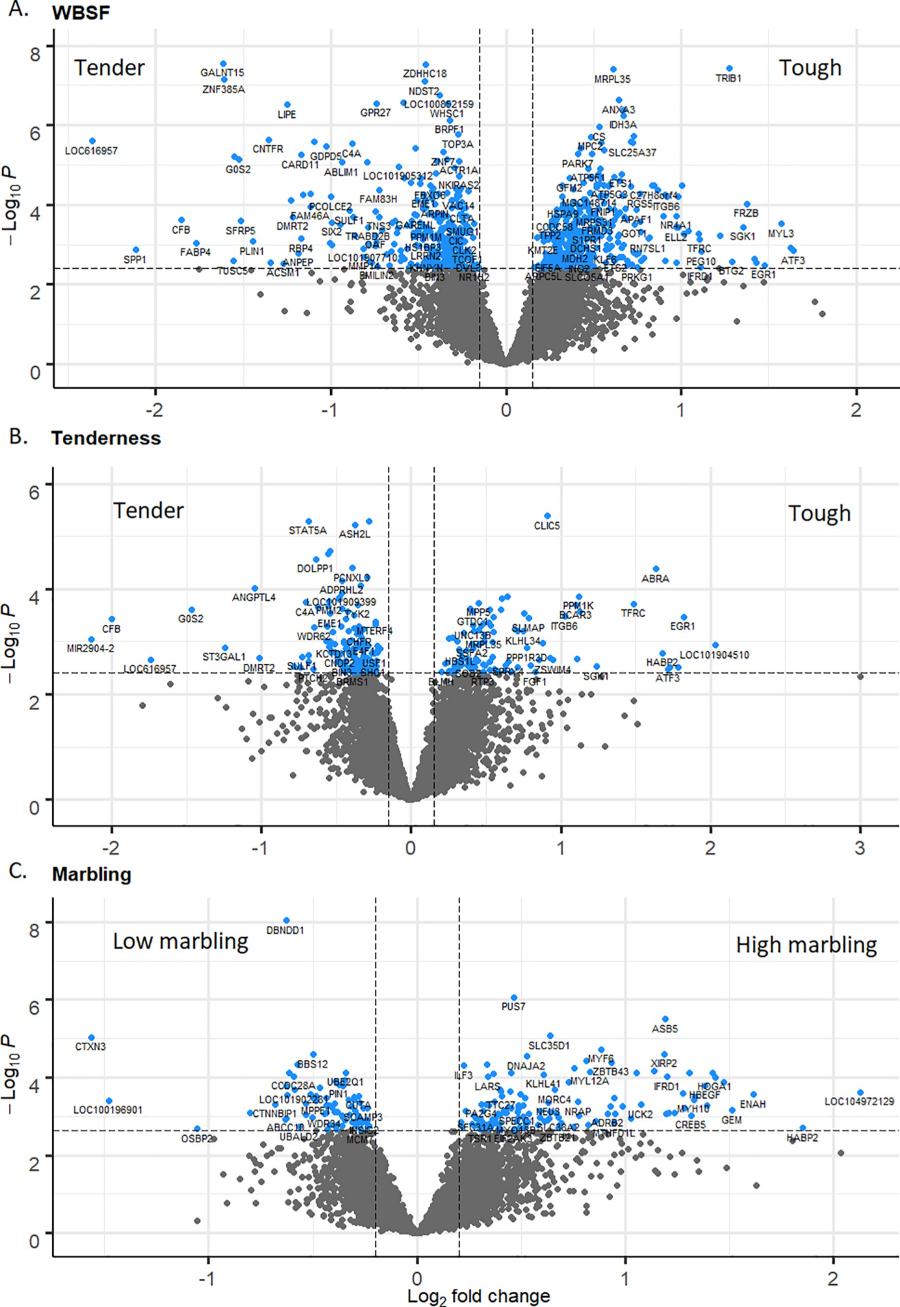
Volcano plots relating log fold change and p-value for WBSF (A), tenderness (B) and marbling (C). Blue dots represent DE genes. A total of 676 (adjusted p-value ≤ 0.05), 70 (adjusted p-value ≤ 0.1) and 198 (adjusted p-value ≤ 0.1) genes were DE for WBSF, tenderness and marbling, respectively.

A total of 676 (Fig 2A; adjusted p-value ≤ 0.05), 70 (Fig 2B; adjusted p-value ≤ 0.1) and 198 (Fig 2C; adjusted p-value ≤ 0.1) genes were DE for WBSF, tenderness and marbling, respectively (S3 Table).

#### Differentially expressed isoforms

**Fig 3:**
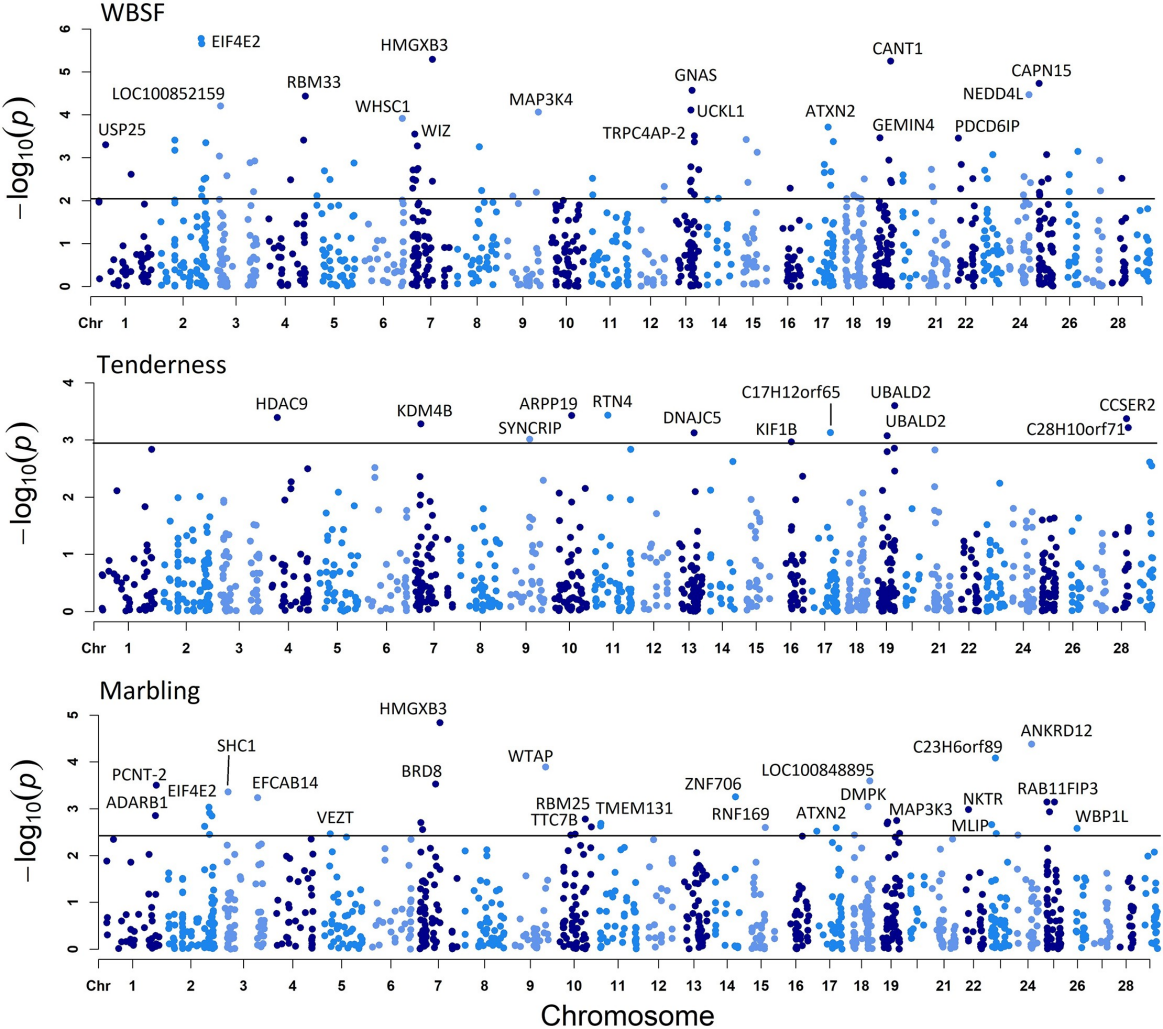
Genes whose isoforms were identified as DE for WBSF (A), tenderness (B) and marbling (C). The x-axis represents gene location across the bovine genome. The black line shows the 0.1 FDR threshold. Different colors represent different chromosomes.

A total of 106 isoforms from 98 genes for WBSF, 13 isoforms from 13 genes for tenderness and 43 isoforms from 42 genes for marbling (Fig 3 and S4 Table; FDR ≤ 0.1) were DE.

### Gene enrichment analysis

A gene enrichment analysis was performed using the four gene lists generated from the expression association and DE gene analyses for WBSF, marbling and tenderness (S5 Table). Ten pathways were identified as enriched and they can be classified into two different groups, pathways associated with cellular structure and pathways associated with respiration.

## Discussion

### Phenotypic data

Similar values related to meat quality parameters have been reported in Brahman and Brahman-influenced populations [55]. For the expression association analysis, animals with low meat quality index had less marbling and more connective tissue, and were tougher and dryer than animals with high index (S1 Fig). For the DE analysis, a clear phenotypic differentiation between high and low performance samples was evident for WBSF, tenderness and marbling.

### Paired-end read alignment and paired-end read counting

Highly specialized genes in skeletal muscle such as *Titin* (*TTN*), *Actin Alpha 1* (*ACTA1*), *Myosin Heavy chain 1* (*MYH1*), *Aldolase Fructose-Bisphosphate A* (*ALDOA*), *Myosin Heavy Chain 7* (*MYH7*), *Nebulin* (*NEB*), *Filamin C* (*FLNC*), *ATPase Sarcoplasmic/Endoplasmic Reticulum Ca2+ Transporting 1* (*ATP2A1*), *Tropomyosin 2* (*TPM2*), and *Creatine Kinase*, *M-type* (*CKM*) were the top expressed genes based on number of counts. Since most of these proteins have structural function and are mechanically required for contraction, they are highly expressed in skeletal muscle. TTN and NEB are large sarcomere filament-binding proteins uniformly expressed in skeletal muscle; NEB acts as an actin filament stabilizer, it is involved in myofibrillogenesis, modulates thin filament length and allows proper muscle contraction [56]. *NEB* knockout mice show muscular weakness, altered calcium homeostasis and glycogen metabolism [56].

### Expression association analysis for the meat quality index

#### Gene expression association analysis

**Fig 4:**
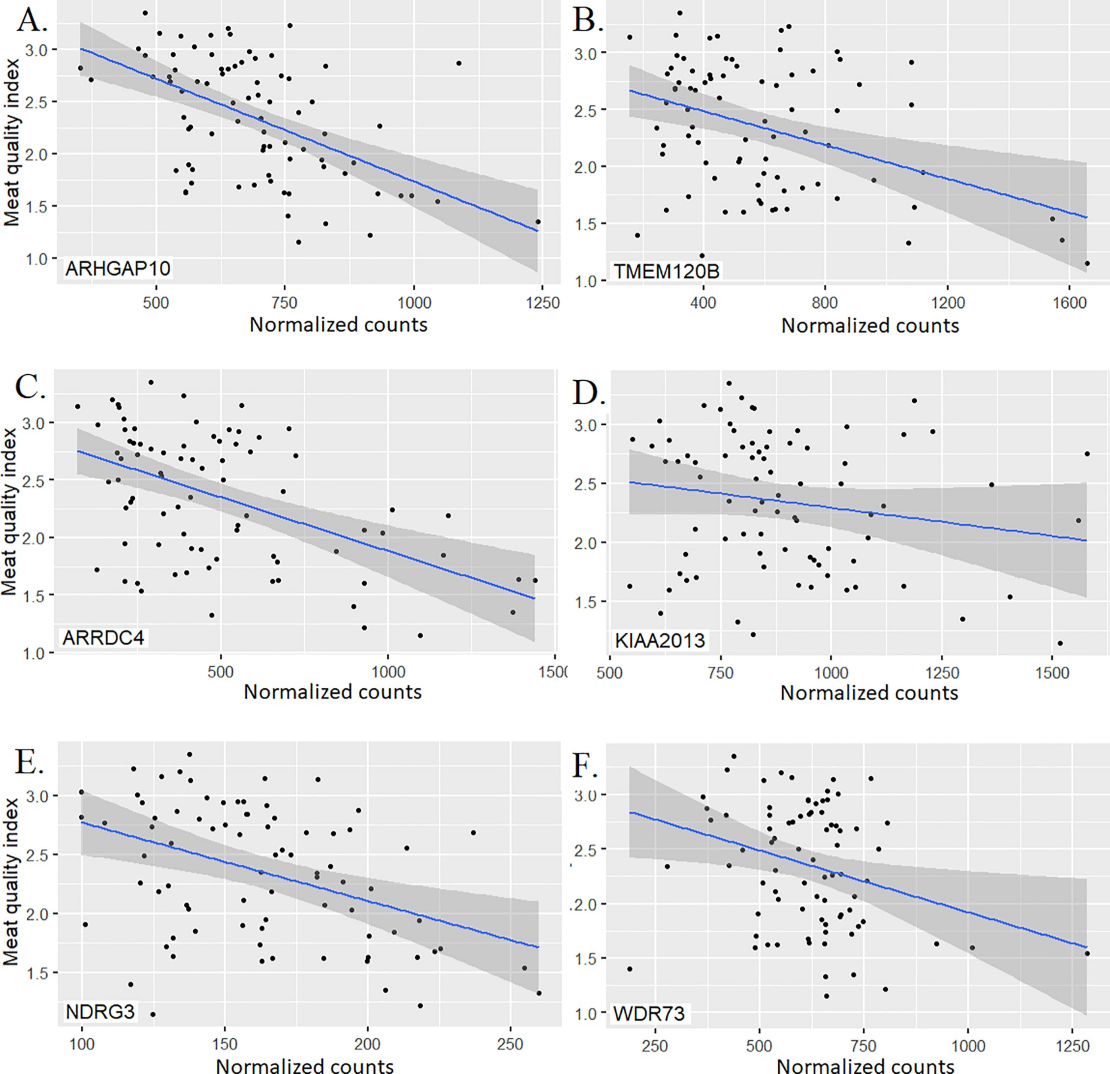
Scatter plots with regression lines and 95% confidence intervals for gene normalized counts and meat quality index for the top six associated genes. The meat quality index was constructed using observed phenotypes measured in *longissimus dorsi* muscle from a multibreed Angus-Brahman population.

In the following paragraphs we present a short description of the most important genes identified through the gene expression association analysis. The gene showing the most significant association (p-value ≤ 4.2x10^-4^), *ARHGAP10* (Fig 4A), is part of a Rho family of GTPase-activating proteins (RhoGAP). This protein regulates the activity of the small GTPase CDC42 and by doing so, controls the F-Actin and ARP2/3 dynamics at the Golgi complex. The Golgi-associated small GTPase, ARF1 recruits ARHGAP21 and allows interaction between ARHGAP21 and CDC42, inducing GTP hydrolysis and promoting actin filament interaction with Golgi membranes [57]. The *ARHGAP10* gene was found to regulate actin cytoskeleton remodeling, cell proliferation, and cell differentiation. The ARHGAP10 interacts with α-tubulin and it is involved in cell-cell adhesion processes and consequently could promote cell migration [58–62]. In our study, overexpression of ARHGAP10 was associated with lower meat quality index. This could be a consequence of a more stable actin cytoskeleton structure which would result in lower meat quality.

A higher expression of *TMEM120B* gene was associated with a reduced meat quality index in the present analysis (Fig 4B). The *TMEM120B* gene is highly expressed during adipocyte differentiation, and knockdown of this gene alters expression of genes required for adipocyte differentiation such as *GATA3*, *FASN* and *GLUT4* [63]. This gene is a cytoskeletal anchoring protein and it can affect tenderness by promoting changes in cytoskeletal structure stability or cellular compartmentalization and size adaptation in adipocytes [64].

The *ARRDC4* gene belongs to a plasma membrane associated protein family named α-arrestins, and higher expression of this gene was associated with lower meat quality index (Fig 4C). A better characterized member of this family, *ARRDC3*, is a breast and prostate cancer suppressor; lower expression of *ARRDC3* was significantly associated with high aggressiveness and metastasis in prostate cancer cells [65,66]. The ARRDC3 protein localizes in certain sections of the plasma membrane associated with intracellular vesicles suggesting that ARRDC3 regulates cell-surface proteins such as ITGβ4 in skeletal muscle; this interaction between ARRDC3 and ITGβ4 suggests a possible mechanism through which ARRDC3 could regulate cell motility and migration [66]. The *ARRDC3* knockout male mouse is resistant to obesity which was reported to be a result of higher energy expenditure due to increased activity level and thermogenesis in adipose tissues [67]. The association of this gene with meat quality could be explained by variation in adipocyte proliferation or overall cytoskeletal structure and cellular attachment.

The *KIAA2013* encodes an uncharacterized transmembrane protein [68] and higher expression of this gene was associated with lower meat quality index (Fig 4D). Xu et al. [69] identified selection signatures on *KIAA2013* using Holstein, Angus, Charolais, Brahman, and N’Dama cattle.

Upregulation of *NDRG3* is present in prostate and laryngeal squamous cancerous cells and was also correlated with pathological stage, positive metastatic status and lymph node status [70–72]. High expression of *NDRG3* was associated with lower meat quality index (Fig 4E), possibly by generating a more stable cellular attachment [73]. This is supported by the fact that upregulation of a *NDRG3* paralogous, *NDRG2*, suppresses tumor invasion by inhibiting the matrix metalloproteinases MMP-9 and MMP-2.

Higher expression of *WDR73* was associated with lower meat quality index (Fig 4F) possibly due to an increment in cytoskeletal structure stability resulting in lower meat quality. Fibroblasts with mutated *WDR73* presented abnormal nuclear morphology, low cell viability, and altered microtubule network, suggesting a role in cellular architecture maintenance and cell survival [74]. Downregulation of *WDR77* arrests growth and differentiation of lung epithelial cells while its upregulation promoted terminally differentiated cells to undergo a new stage of cell proliferation, triggering lung adenocarcinoma formation [75].

#### Exon expression association analysis

**Fig 5:**
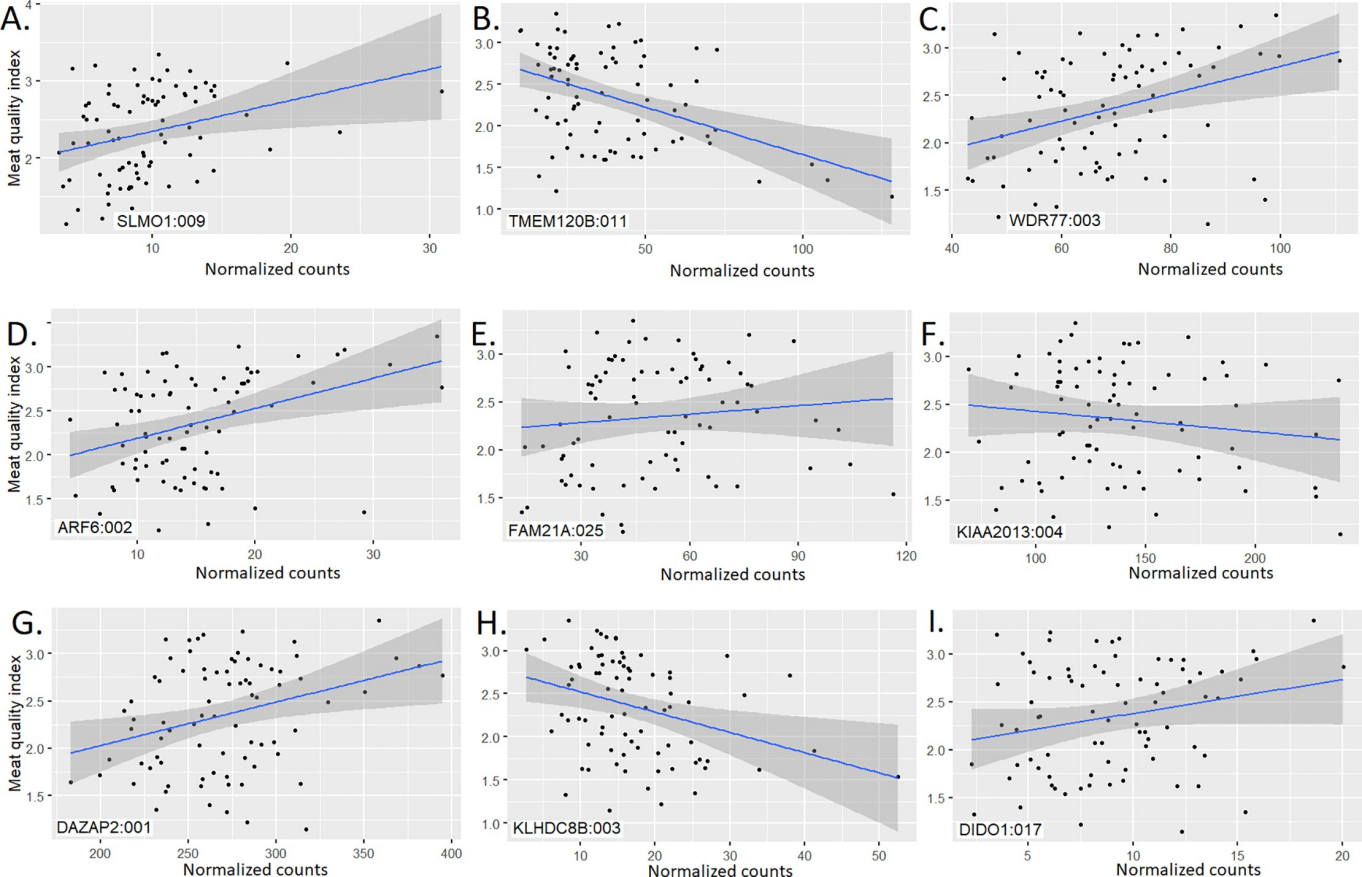
Scatter plots with regression lines and 95% confidence intervals for exon normalized counts and meat quality index for the top nine associated genes.

The *SLMO1*, *TMEM120B*, *ARF6*, *FAM21A*, *KIAA2013*, *DAZAP2*, *KLHDC8B* and *DIDO1* genes are discussed below. The *SLMO1* gene encodes three different isoforms (S6 Table) and two of them share exon 9. Higher expression of the exon 9 of *SLMO1* was associated with higher meat quality index (Fig 5A). This association could be due to increased lipid deposition given that this protein is part of an intermembrane lipid transfer system located in the mitochondria [76] or it could contribute to cytoskeletal attachment of this organelle membrane. The exon 9 of SLMO1 encodes a total of 30 amino acids (golden region in the S2 Fig) that are part of a PRELI/MSF1 domain. This domain is located between positions 74 and 245 and confers a globular alpha-beta folded structure to SLMO1 [77]. The association of the exon 9 with meat quality index shows that the isoforms ENSBTAT00000081878.1 and ENSBTAT00000046981.3 could have a similar phenotypic effect on meat quality in the present population but different from the effect of the isoform ENSBTAT00000084244.1.

Expression of multiple exons of the *TMEM120B* gene and the exon 3 of *WDR77* agreed with the overall gene expression association analysis (Fig 5B and 5C). All *TMEM120B* exons were individually associated with the meat quality index. The exon 3 in *WDR77* encodes a segment between the amino acids 99 and 148 located inside the WD_REPEATS_REGION which could be important for the formation of the globular structure shown in the S3 Fig (golden region).

Higher expression of the exon 2 of *ARF6* was associated with higher meat quality index (Fig 5D) probably due to cell proliferation and cytoskeletal remodeling. This gene encodes a GTP-binding protein involved in plasma membrane trafficking, actin-based cytoskeletal remodeling and cell migration [78]. Knockout *ARF6* mice exhibit hypocellularity, midgestational hepatocyte apoptosis with Caspase 3 activation, defective hepatic cord formation and almost completely penetrant embryonic lethality [79].

Higher expression of the exon 25 of *FAM21A* was associated with higher meat quality index (Fig 5E); *FAM21A* interacts with a multi-protein complex named WASH (Wiskott-Aldrich Syndrome Protein and SCAR Homolog) involved in endosome-to-plasma membrane trafficking. This complex interacts with tubulin and F-actin, and activates ARP2/3, and endocytosis, sorting and trafficking regulator [80]. The association of FAM21 and meat quality could be due to changes in actin polymerization. The FAM21 protein modulates actin polymerization by preventing actin-capping through a physical interaction with the Capping Actin Protein of Muscle Z-Line (CAPZ). Additionally, FAM21 can interact with phosphatidylserine and some phospholipid species allowing the linkage between the WASH complex and endosomal domains [81,82].

The third and fourth exons of *KIAA2013* were associated with meat quality index (Fig 5F) and higher expression of both were associated with lower meat quality index. This gene encodes an uncharacterized transmembrane protein [68] showing that this protein could be a cytoskeletal anchor. Two different transmembrane regions were predicted between the positions 21–40 and 592–614; the latter transmembrane region is encoded by the third *KIAA2013* exon (S6 Table).

Higher expression of the first exon of *DAZAP2* was associated with higher meat quality index (Fig 5G) and this relationship could be due to cell proliferation given that this gene is a potential tumor suppressor. Patients with multiple myeloma have *DAZAP2* downregulation because of promoter methylation [83,84].

Meat quality index was negatively correlated with expression of the third exon of *KLHDC8B* (Fig 5H) and this gene is associated with some cases of classical Hodgkin lymphoma which is characterized by binucleated cells. The *KLHDC8B* gene encodes a midbody kelch protein required during mitotic cytokinesis [85,86]. The third *KLHDC8B* exon is included in both annotated isoforms (S4 Fig) suggesting that additional isoforms involving this exon may be still uncovered. The third *KLHDC8B* exon could be structurally important for providing a globular conformation (golden region).

Expression of the exon number 17 of *DIDO1* was associated with higher meat quality index (Fig 5I). Two different *DIDO1* isoforms are annotated but only the isoform ENSBTAT00000007879.6 includes the exon 17 (S6 Table). This isoform has an additional domain located between the amino acids 672 and 792 (TFIIS_CENTRAL) involved in mRNA cleavage [77]. The protein segment encoded by the exon number 17 of DIDO1 (from amino acid number 1088 to 1117) could be structurally crucial for overall molecular activity. The association between exon expression and meat quality index could be related to the pro-apoptotic activity of *DIDO1* [87]. DIDO1 is involved in regulating embryonic stem cell maintenance and there exists early differentiation in mouse embryonic stem cells lacking this gene; this protein is also able to positively regulate expression of key pluripotency markers [88].

### Differential expression analysis

#### DE genes for WBSF, tenderness and marbling

A total of 19 genes were simultaneously identified in at least two analyses and they can be classified into three different groups based on their biological function. The first group of DE genes are related to cell survival, apoptosis and cancer, and include the following genes:

*Angiopoietin Like 4* (*ANGPTL4*), *Apoptotic Peptidase Activating Factor 1* (*APAF1*), *G0/G1 Switch 2* (*G0S2*), *Hyaluronan Binding Protein 2* (*HABP2*), *Interferon Related Developmental Regulator 1* (*IFRD1*) and *Tribbles Pseudokinase 1* (*TRIB1*). These genes could promote myocyte and adipocyte proliferation. The second group includes a number of structural proteins associated with cellular membranes or cytoskeletal proteins. The genes.

*Complement C4A* (*C4A*), *Complement Factor B* (*CFB*), *Chloride Intracellular Channel 5* (*CLIC5*), *Family With Sequence Similarity 83 Member H* (*FAM83H*), *Integrin Subunit Beta 6* (*ITGB6*), *Mitochondrial Ribosomal Protein L35* (*MRPL35*), *Phospholamban* (*PLN*), *Protein Phosphatase*, *Mg2+/Mn2+ Dependent 1K* (*PPM1K*), *Transferrin Receptor* (*TFRC*), *Tripartite Motif Containing 55* (*TRIM55*) belong to this group. Changes in the amount of these proteins could have a direct effect on cytoskeletal structure and organization, and postmortem proteolysis. Two transcription factors, *Early Growth Response 1* (*EGR1*) and *Hes Related Family BHLH Transcription Factor with YRPW Motif-Like* (*HEYL*), were also uncovered and they represent the third group. The most important DE genes associated with meat quality in the present analysis are described below.

The *APAF1* gene was identified as DE in the WBSF and tenderness analyses, and was identified as downregulated in tender meat; this protein is a central component of the apoptosome, a mitochondrial caspase activation pathway which mediates apoptosis. After activation of this pathway, the mitochondria release Cytochrome C which in turn binds to APAF1 and promote apoptosis by activating Caspase 9 [89,90]. Long et al. [90] characterized an *APAF1* mutant mouse line which does not promote apoptosis. These mouse embryos presented decreased apoptosis, nervous system development defects and craniofacial deficiencies associated with higher mesenchymal proliferation and delayed ossification resulting in perinatal death. In humans, downregulation of *APAF1* is evident in colorectal cancer and hepatocellular carcinoma cells given transcriptional regulation by miR-23a and Histone Deacetylases 1–3 [89,91].

The *G0S2* gene was upregulated in tender meat in the WBSF and tenderness analyses; *G0S2* is highly expressed in adipose tissue and its expression relates to lipid accumulation and adipogenesis in swine. Cell proliferation inhibition is also promoted by this gene giving that there exit *G0S2* downregulation in preadipocytes and fetal adipose tissues, and upregulation in adipocytes and adipose tissues from adult pigs [92]. Lipid catabolism is regulated by G0S2 through interaction and inhibition of the Adipose Triglyceride Lipase (ATGL) and upregulation of *G0S2* or downregulation of *ATGL* in non-small cell lung carcinomas stalls triglyceride catabolism and represses cell growth [93]. Female knockout *G0S2* mice present lactation defects and knockout mice show lower body weight gain, higher serum glycerol levels, higher acute cold tolerance given upregulation of thermoregulatory and oxidation promoting genes in white adipose tissue [94,95]. High *G0S2* methylation is present in squamous lung cancer being this methylation content inversely correlated with *G0S2* expression [96].

The *IFRD1* gene was downregulated in tender meat in the WBSF analysis; however, this gene was upregulated in high marbling samples. *IFRD1* plays a role in muscle differentiation and bone homeostasis. In myoblasts, downregulation of *IFRD1* hinders cell cycle exit and differentiation via MyoD downregulation, and promotes acetylation and nuclear localization of p65. In adult muscle, upregulation of *IFRD1* stimulates regeneration via myogenesis by negatively regulating NF-κB, which in turn is post-transcriptionally downregulate by MyoD [97]. In bone, *IFRD1* is involved in bone homeostasis maintenance; knockout *IFRD1* mice develop higher bone mass because of increased bone deposition and decreased bone reabsorption [98].

Downregulation of *CLIC5* was identified in tender meat in the WBSF and tenderness assessment. This gene encodes a multiconductance channel for Na+, K+ and Cl–, and is inactivated by F-actin; this channel modulates solute transport at key cellular stages such as apoptosis, and cell division and fusion [99]. A CLIC5 isoform, CLIC5A, is involved in glomerular endothelial cell and podocyte architecture formation and maintenance, and both cell types show high *CLIC5A* expression. This isoform colocalized with Podocalyxin (PODXL) and Ezrin (EZR) in the apical plasma membrane in podocytes. Knockout *CLIC5A* mice present lower *EZR* expression in podocytes altering PODXL and actin filament association [100]. Berryman, Bruno, Price, & Edwards [101] reported that the *de novo* assembly of the cytoskeletal complex CLIC5A-EZR requires actin polymerization, being CLIC5A essential for assembly and maintenance of F-actin-based arrangement at the cell cortex.

Upregulation of *FAM83H* was identified in tender meat using the WBSF and tenderness analyses. FAM83H colocalizes with keratin filaments surrounding the nucleus and usually communicates with cell-cell junctions. Downregulation of *FAM83H* promotes keratin filament formation and its upregulation produces keratin filament disassembly. The filamentous keratin structure is regulated by FAM83H and disorganization of this keratin associated cytoskeleton is caused by upregulation of *FAM83H* in colorectal cancer cells [102]. Upregulation of *FAM83H* is mediated by binding of MYC at *FAM83H* promoter and is characteristic of hepatocellular carcinoma cells. Overexpression of *FAM83H* drives upregulation of *Cyclin D1*, *Cyclin E1*, *SNAI1* and *MMP2*, and repression of *P53* and *P27* [103].

Downregulation of *PLN* in tender meat was identified using the WBSF analysis; however, this gene was upregulated in high marbling samples. PLN is a sarcoplasmic reticulum Ca2+-cycling protein and regulatory partner of the ATPase Sarcoplasmic/Endoplasmic Reticulum Ca2+ Transporting 2 (ATP2A2) protein being involved in regulating cardiomyocyte contractility [104,105]. Medin et al. [106] identified a SNP located in the promoter region of *PLN* able to decrease its transcriptional activity and associated with apical hypertrophic cardiomyopathy. Some mutations in the cytoplasmic domain of PLN modify its hydrophobic interaction with ATP2A2, and alter PLN regulatory activity. One of these mutations, a deletion in the coding region is associated with left ventricular dilation, contractile dysfunction, episodic ventricular arrhythmias and hereditary heart failure. Transgenic mice overexpressing the *PLN*-Del allele develop similar symptomatology as well as premature death [104]. This PLN deletion abolishes regulation by phosphorylation, which in turn induces a constitutive PLN inhibitory state [107].

The directionality of expression of most of these genes agreed across analysis. The expression of *CFB*, *G0S2*, *C4A*, *ANGPTL4* and *FAM83H* was higher in tender meat and expression of *MRPL35*, *CLIC5*, *KLHL34*, *HEYL*, *APAF1*, *ITGB6*, *PPM1K*, *TFRC*, *TRIB1* and *EGR1* was lower in tender meat.

#### Isoform DE analysis for WBSF, tenderness and marbling

Because isoform annotation for the Btau_4.6.1 reference genome is relatively poor, only gene name in the isoform association analysis was reported and further evaluation was carried out for well annotated isoforms. The *Eukaryotic Translation Initiation Factor 4E Family Member 2* (*EIF4E2*), *GNAS Complex Locus* (GNAS), *Lysosomal Associated Membrane Protein 2* (LAMP2), *Mucolipin 1* (MCOLN1) and *Reticulon 4* (*RTN4*) genes were selected for further analysis.

The *EIF4E2* isoform NM_001075795.2 was identified as DE (S7 Table). Hypoxic microenvironment is a common feature in tumors and EIF4E2 is preferentially used rather than EIF4E during translation of a number of genes [108] such as cytoskeletal related proteins. Cadherin-22 is a cell-surface molecule target of EIF4E2 and it is involved in cell migration, invasion and adhesion during cancer development. Kelly et al. [109] reported that silencing of EIF4E2 or Cadherin-22 halted breast carcinoma and glioblastoma development during hypoxia. The EIF4E2 isoforms NP_001069263.1 and NP_001193345.1 only differ by a 12-amino acid segment (golden region in S5 Fig). The additional segment present in NP_001069263.1 could confer a differential effect on EIF4E2 translational function during apoptosis affecting the tenderization process.

The S7 Table shows some structural features of the GNAS isoforms NP_001258700.1 and NP_851364.1, being the latter isoform identified as DE in the present analysis. Both isoforms differ greatly because of alternative promoters. The GNAS locus is paternally, maternally and biallelically imprinted in a tissue-specific manner and code for a number of molecular products by using multiple promoters [110]. The GNAS protein is categorized as a cell membrane associated protein [68], thus it could contribute to cytoskeletal stability. Furukawa et al. [111] and Wu et al. [112] reported that somatic mutations in the GNAS locus are frequently identified in Intraductal papillary mucinous neoplasm, a pancreatic cystic neoplasm characterized by being highly invasive and metastatic with poor prognosis. Markers in the GNAS locus are also associated with endocrine tumors, fibrous dysplasia of bone and hereditary osteodystrophy [110].

Isoforms from the *LAMP2* and *MCOLN1* genes were identified as DE, and their proteins are lysosomal associated proteins. For the LAMP2 gene, the NP_001029742.1 and NP_001106715.1 isoforms were analyzed. The NP_001106715.1 (homologous to the LAMP2A isoform in mice) was determined as DE in the present population. Both isoforms have a signal peptide and two transmembrane segments (S7 Table) nevertheless, homology between them decreases after the amino acid number 363. A monomeric LAMP2A molecule binds to substrate proteins and allows chaperone-mediated autophagy in lysosomes by establishing high-molecular-weight LAMP2A complexes at the lysosomal membrane; the hsc70 and hsp90 chaperones have crucial roles in disassembly and stabilization of the LAMP2A complexes [113]. Cuervo & Dice [114] found that 25% of total LAMP2 molecules in rat liver lysosomes were LAMP2A and concentration of this isoform was correlated with rates of chaperone-mediated autophagy in liver and fibroblasts in culture; therefore, there exists a substrate protein that binds only to the LAMP2A isoform. The LAMP2A isoform also mediates autophagosome-lysosome fusion in mouse embryonic fibroblasts [115]. The MCOLN1 isoform NP_001159604.1 was identified as DE (S7 Table). This protein is a Ca2+-releasing cation channel associated with the lysosomal plasma membrane and it is involved in endocytosis. Mutations in this gene cause mislocalization and disrupt Ca2+ flow across the lysosomal membrane and produce Mucolipidosis type IV, a lysosomal storage disorder related to a transport defect in endocytosis [116,117]. Schmiege et al. [117] reported the conformational assembly of the human MCOLN1 channel which is structurally close to the bovine isoform NP_001159604.1 (S6 Fig); this channel seems to be tightly regulated by aromatic–aromatic and hydrophilic interactions between amino acids and by agonist regulation, allowing adequate selectivity filter dynamics. Cuajungco et al. [118] reported physical interaction between MCOLN1 and TRPML1, a zinc transporter, and deletion of the MCOLN1’s N-terminus disrupted this interaction. Some other mutations in this gene are able to disrupt inhibition of MCOLN1 by pH and promote channel aggregation [119]. Expression of the DE isoforms of *LAMP2* (NM_001113244.1) and *MCOLN1* (NM_001166132.1) could promote specific cytoskeletal association with lysosome membranes. This effect on cytoskeletal organization may contribute to overall tenderization postmortem and meat quality.

The RTN4 isoform NP_001106692.1 was identified as DE in the present analysis (S7 Table); this isoform is homologous to the human RTN4 isoform B. RTNs encode a family of membrane associated proteins and RTN4s are involved in shaping and maintaining endoplasmic reticulum tubules. RTN4, Atlastin (ATL) and Lunapark, ER Junction Formation Factor (LNP) proteins are curvature-stabilizing proteins required for the formation of the cellular network of membrane tubules and a RTN4/ATL activity balance is required. Hyperactivity or upregulation of RTN4A induces endoplasmic reticulum fragmentation [120,121]. The RNT4B is expressed in epithelial, fibroblast and neuronal cells and it is localized in curved membranes on endoplasmic reticulum tubules and sheet edges. Upregulation of RNT4B modifies the sheet/tubule balance and induces higher formation of tubules producing membrane deformation; conversely, RNT4B downregulation produces large peripheral endoplasmic reticulum sheets [122].

### Overlapping genes across DE evaluation and genome wide association analysis in the present population

**Fig 6:**
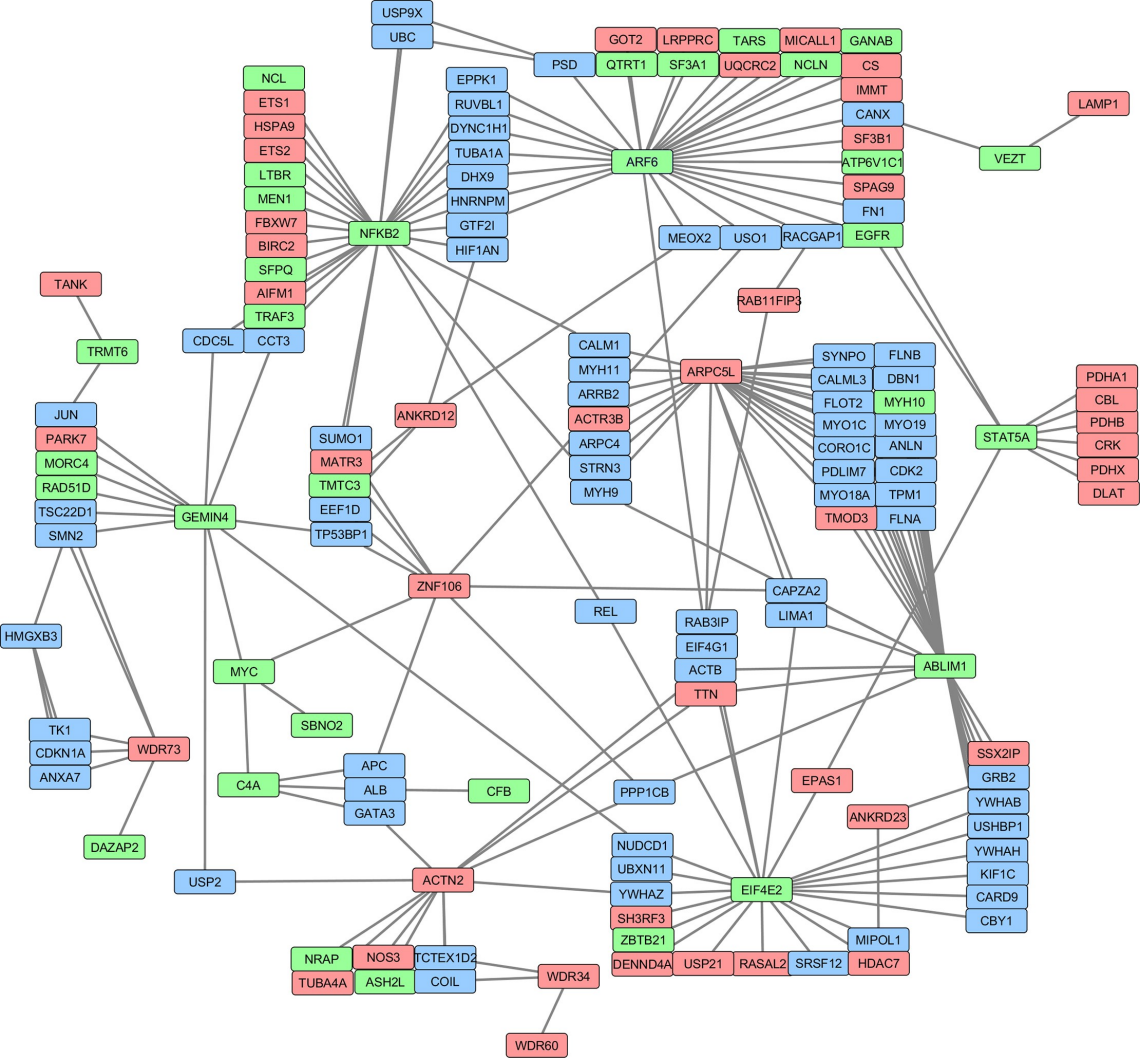
Protein-protein interaction network showing upregulated (green) and downregulated (red) genes in tender meat from *longissimus dorsi* muscle sampled in a multibreed Angus-Brahman population. Blue boxes show genes that were not identified in the expression or DE analysis but are part of the network.

A total of 30 genes were simultaneously identified in the expression association and DE analysis, and 13 of them encode proteins with structural function; five other genes are transcription factors or co-regulators (Table 2). From the structural proteins, 12 are potential μ-calpain substrates. All 30 genes were initially used to construct a protein-protein interaction network (Fig 6). Out of these 30 genes, 18 genes constitute a network including 150 proteins. From these 150 proteins, 45 were determined as downregulated (red nodes) and 31 others as upregulated (green nodes) in tender samples. Other 78 genes (blue nodes) were not identified in the expression or DE analysis but interconnect with other nodes of this protein-protein interaction network. In this network, *NFKB2* (upregulated), *ABLIM1* (upregulated), *EIF4E2* (upregulated) and *ARPC5L* (downregulated), and *ARF6* (upregulated) showed the highest connectivity.

**Table 2:** Genes that were identified at least three times using the expression association and DE analysis approach for meat quality related phenotypes. Meat quality was recorded in *longissimus dorsi* muscle from a multibreed Angus-Brahman population.

Gene	Expression		DE			Isoform		Function
name	Gene	Exon*	WBSF	Tenderness	Marbling	WBSF	Marbling	
*ABLIM1+*	X	X	X					Cytoskeleton
*ACTN2+*			X	X	X			Cytoskeleton
*ANKRD12*	X					X	X	
*ANKRD23*			X			X	X	
*ARPC5L+*	X	X	X					Cytoskeleton
*C4A+*	X	X	X	X				Membrane
*CFB+*		X	X	X				Membrane
*EIF4E2*			X			X	X	RNA binding
*GEMIN4*			X			X	X	
*HMGXB3*		X				X	X	Transcription
*LOC100852159*			X	X		X		
*LOC101903649*			X	X		X		
*MON1B*		X	X	X				
*MPPE1+*	X	X			X			Cytoskeleton
*NFKB2*	X	X			X			Transcription
*PCNXL3*		X	X	X				
*PCOLCE2*	X	X	X					Peptidase regulation
*SBNO2*		X	X	X				Transcription
*ST6GALNAC2+*	X	X			X			Membrane
*STAT5A*	X	X	X	X				Transcription
*TMEM131+*		X				X	X	Membrane
*TRMT6*	X	X			X			
*UCP2+*	X	X	X					Membrane
*UNC13B*	X	X		X				
*VEZT+*	X	X					X	Membrane
*WBP1L*			X			X	X	
*WDR34+*	X	X			X			Cytoskeleton
*WDR73+*	X	X			X			Cytoskeleton
*ZNF106*	X	X		X				
*ZNF771*	X	X			X			Transcription

* Genes with at least three associated exons were included.

+ The protease analysis was carried out using the PROSPER server [80].

The key genes identified in the protein-protein interaction network (Fig 6), *NFKB2*, *ABLIM1*, *EIF4E2*, *ARPC5L* and *ARF6*, are involved in multiple cellular functions such as actin polymerization, cytoskeletal structure and transcription factor activity [68].

Table 3 shows a list of genes that were simultaneously identified by Leal-Gutiérrez et al. (2018c) [123] and Leal-Gutiérrez et al. [7] using genotype-phenotype association in the present population and genes that were identified in the expression or DE analysis. A total of 14 genes were identified using genotype-phenotype and expression-phenotype association approaches simultaneously. These genes could potentially exhibit cis-eQTL regulation suggesting that changes in gene expression could be responsible for the genotype-phenotype association. Based on this theory, a cis-eQTL analysis was performed (unpublished data). Cis-eQTL regulation was identified for the *3-Hydroxyisobutyrate Dehydrogenase* (*HIBADH*) and *SRSF Protein Kinase 1* (*SRPK1*). This result suggests that polymorphisms in these genes could be able to regulate the expression of harboring genes, and this variation in mRNA expression could have a direct effect on meat quality in the present population.

**Table 3:** Genes uncovered by the expression association and DE analysis, and associated with meat quality.

Analysis	SEM analysis	GWAS Analysis
Expression	SRPK1*	* *
Expression	*ZFYVE26*	* *
Expression	* *	*LRP5*
DE genes	*ZNF385A*	* *
DE genes	*NCOA5*	* *
DE genes	*BOD1L1*	* *
DE genes	HIBADH*	* *
DE genes	* *	*GOSR2*
DE genes	*KDR*	* *
DE genes	*ATPAF1*	* *
DE genes	*ZBTB39*	* *
DE Isoforms	*EIF4ENIF1*	* *
DE Isoforms	*EFCAB14*	* *
DE Isoforms	*RTN4*	* *

The association analysis was performed in the same population (81,82).

* Genes with Cis-eQTL regulation identified in the present population.

The phenotypes were recorded in *longissimus dorsi* muscle from a multibreed Angus-Brahman population. * Genes with cis-eQTL effects (unpublished data).

### Gene enrichment analysis

The ten enriched pathways identified can be classified into two groups. The first group relates to Membrane (GO:0016020) and Membrane part (GO:0044425) which cluster some structural genes. Enrichment of structural protein pathways such as Endoplasmic reticulum membrane (GO:0005789), Golgi apparatus (GO:0005794), and Mitochondrial inner membrane (GO:0005743) were also identified using a gene enrichment analysis based on GWA analysis in the present population [7]. Moreover, enrichment for related pathways such as Cell adhesion and maintenance, Plasma membrane, Integral to plasma membrane, Transmembrane transport, Integral to organelle membrane, Endoplasmic reticulum membrane, and Mitochondrial matrix were identified using copy number variation and selection signatures in Hanwoo, Holstein, Angus, Charolais, Brahman, and N’Dama cattle [69,124]. The second type of pathway, is related to energy metabolism and includes pathways such as Respirasome (GO:0070469), Mitochondrial respiratory chain complex I (GO:0005747) and Respiratory chain complex I (GO:0045271). [69] reported enrichment for ATPase activity and Glucose metabolic process in Holstein, Angus, Charolais, Brahman, and N’Dama.

## Conclusions

Several genes encoding cytoskeletal proteins and transmembrane anchoring molecules were identified in the expression association and DE analysis in the present population and these proteins can have a direct effect on tenderness and marbling. Cytoskeletal proteins and transmembrane anchoring molecules can influence meat quality by allowing cytoskeletal filament interaction with myocyte and organelle membranes, contributing to cytoskeletal structure, microtubule network stability, and cellular architecture maintenance postmortem. Some of these cytoskeletal and transmembrane proteins can modulate cell proliferation. Several pathways related to structural proteins and energy metabolism were identified as enriched showing that these kinds of genes are overrepresented and are crucial for meat quality in the present population.

## Supporting information

S1 Fig Relationship between the meat quality index and observed meat quality related phenotypes. (PNG)

S2 Fig Ribbon representation of the analyzed SLMO1 isoforms. A. ENSBTAT00000081878.1, B. ENSBTAT00000046981.3 and C. ENSBTAT00000084244.1. The exon 9 (located between the amino acids 141 and 170) was identified in the exon expression analysis for the meat quality index and is represented by the golden segment. The models were constructed using the SwissModel server [46–48].
(PNG)

S3 Fig Ribbon representation of the analyzed WDR77 isoform ENSBTAT00000018753.4. The exon 3 (located between the amino acids 99 and 148) was identified in the exon expression analysis for the meat quality index and is represented by the golden segment. B and C represent the ENSBTAT00000018753.4 isoform molecular surface and the golden region denotes the molecular surface of the exon 3. The models were constructed using the SwissModel server [46–48].
(PNG)

S4 Fig Ribbon and molecular surface representation of the analyzed *KLHDC8B* isoforms ENSBTAT00000001298.3 and ENSBTAT00000001299.4. A. Ribbon representation of the analyzed *KLHDC8B* isoform ENSBTAT00000001298.3. The exon 3 (located between the amino acids 126 and 180) was identified in the exon expression analysis for the meat quality index and is represented by the golden segment. B and C represent the ENSBTAT00000001298.3 isoform molecular surface and the golden region denotes the molecular surface of the exon 3. D. Ribbon representation of the analyzed *KLHDC8B* isoform ENSBTAT00000001299.4. The exon 3 (located between the amino acids 126 and 180) is represented by the golden segment. E and F represent the ENSBTAT00000001299.4 isoform molecular surface and the golden region denotes the molecular surface of the exon 3. The models were constructed using the SwissModel server [46–48].
(PNG)

S5 Fig Comparison between the EIF4E2 isoforms NP_001069263.1 and NP_001193345.1. Blue and golden segments were modeled using the SwissModel server [46–48]. The molecular surface of the EIF4E2 isoforms NP_001069263.1 (B) and NP_001193345 (C) are presented and the golden region denotes the additional protein segment present in NP_001193345. The models were constructed using the SwissModel server [46–48].
(PNG)

S6 Fig A. Comparison between the MCOLN1 isoforms NP_001159604.1 and NP_001068690.1. Blue, red and golden regions were modeled; red regions represent transmembrane segments. B. Ribbon representation of the analyzed MCOLN1 isoform NP_001159604.1. C and D represent the NP_001159604.1 isoform molecular surface and the golden region denotes the additional protein segment present in this isoform. E. Ribbon representation of the MCOLN1 isoform NP_001068690.1. F and G represent the NP_001068690.1 isoform molecular surface.
(PNG)

S1 Table Total number of reads, overall read mapping rate and mean paired read fragment inner distance. (XLS)

S2 Table List of genes and exons identified using the expression analysis for the meat quality index. The meat quality index was constructed using observed phenotypes measured in *longissimus dorsi* muscle from a multibreed Angus-Brahman population. The association p-values and estimates of betta for gene and exon normalized counts are shown.
(XLS)

S3 Table List of DE genes for WBSF, tenderness and marbling. The meat quality traits were measured in *longissimus dorsi* muscle from a multibreed Angus-Brahman population. The estimated fold change and adjusted p-value by gene are shown. * Comparison tough vs tender; + comparison high marbling vs low marbling.
(XLS)

S4 Table List of genes which isoforms were determined as DE for WBSF, tenderness and marbling. The meat quality traits were measured in *longissimus dorsi* muscle from a multibreed Angus-Brahman population. The adjusted p-value by gene is shown. Only genes with FDR ≤ 0.05 were included.
(XLS)

S5 Table List of enriched GO terms for the expression and DE gene analysis for WBSF, tenderness and marbling. The meat quality traits were measured in *longissimus dorsi* muscle from a multibreed Angus-Brahman population. The enrichment p-value, number of genes in the pathway (n.anno) and term definitions are shown.
(XLS)

S6 Table Structural features of genes and isoforms identified using the exon expression analysis for the meat quality index. Meat quality was recorded in *longissimus dorsi* muscle from a multibreed Angus-Brahman population. **+** The transmembrane region was predicted using the TMHMM2.0 server [50]; * Protein domains were predicted using the PROSITE server [51]. NA = not applicable.
(XLS)

S7 Table Structural features of selected DE isoforms for WBSF, tenderness and marbling. Meat quality was recorded in *longissimus dorsi* muscle from a multibreed Angus-Brahman population. **^** The signal peptide region was predicted using the SignalIP 4.0 server [52]; **+** the transmembrane regions were predicted using the TMHMM2.0 server [50]; * Protein sequence modeled using the SwissModel server [46–48]. NA = not applicable.
(XLS)
