# Supplementary figures and images for: RNA-seq analysis identifies cytoskeletal structural genes and pathways for meat quality in beef
Source: PLoS One. 2020 Nov 11;15(11):e0240895. doi: 10.1371/journal.pone.0240895 (PMC7657496; doi:10.1371/journal.pone.0240895)

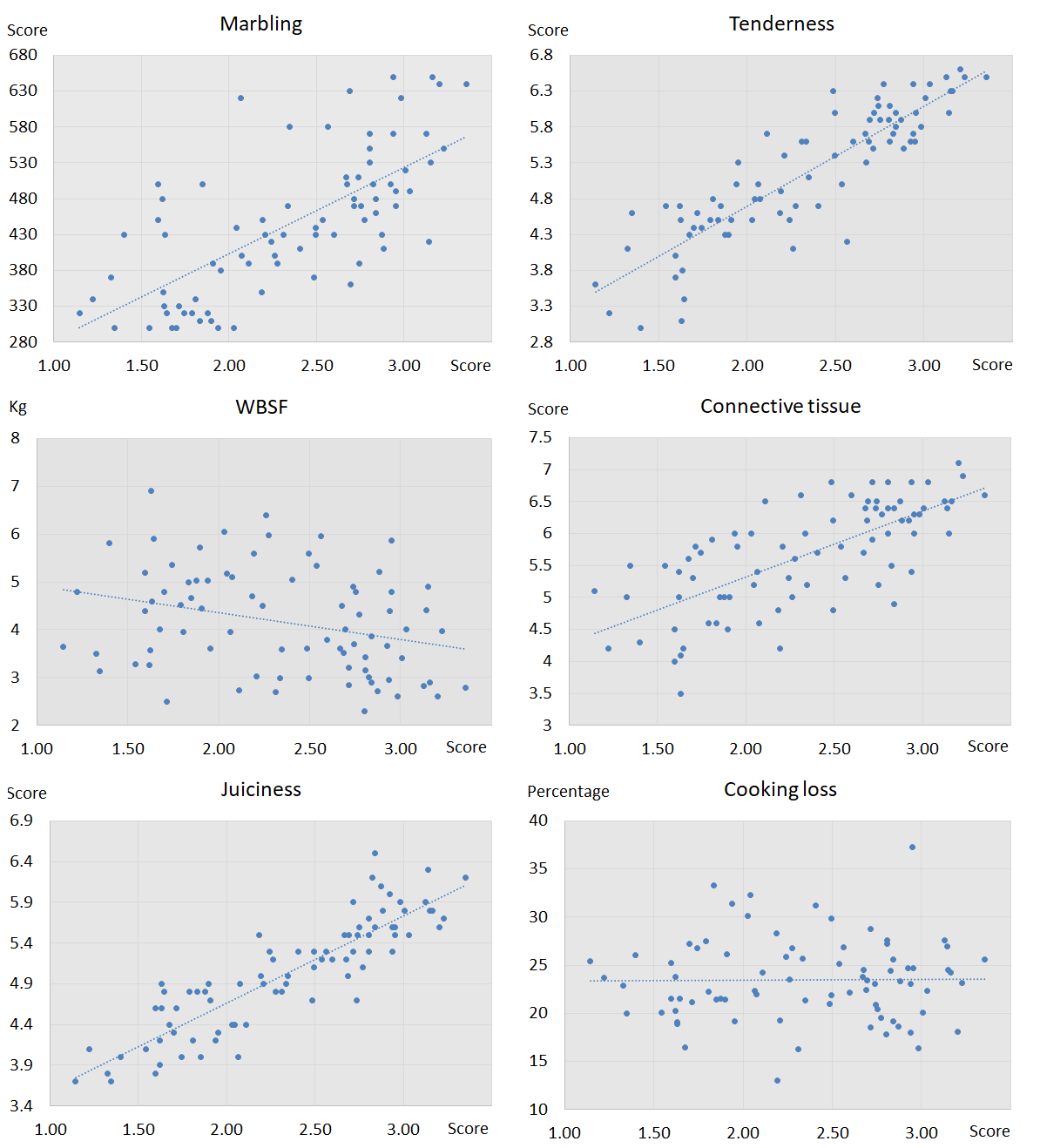

Supplement: S1 Fig — (PNG) [file pone.0240895.s001.png]

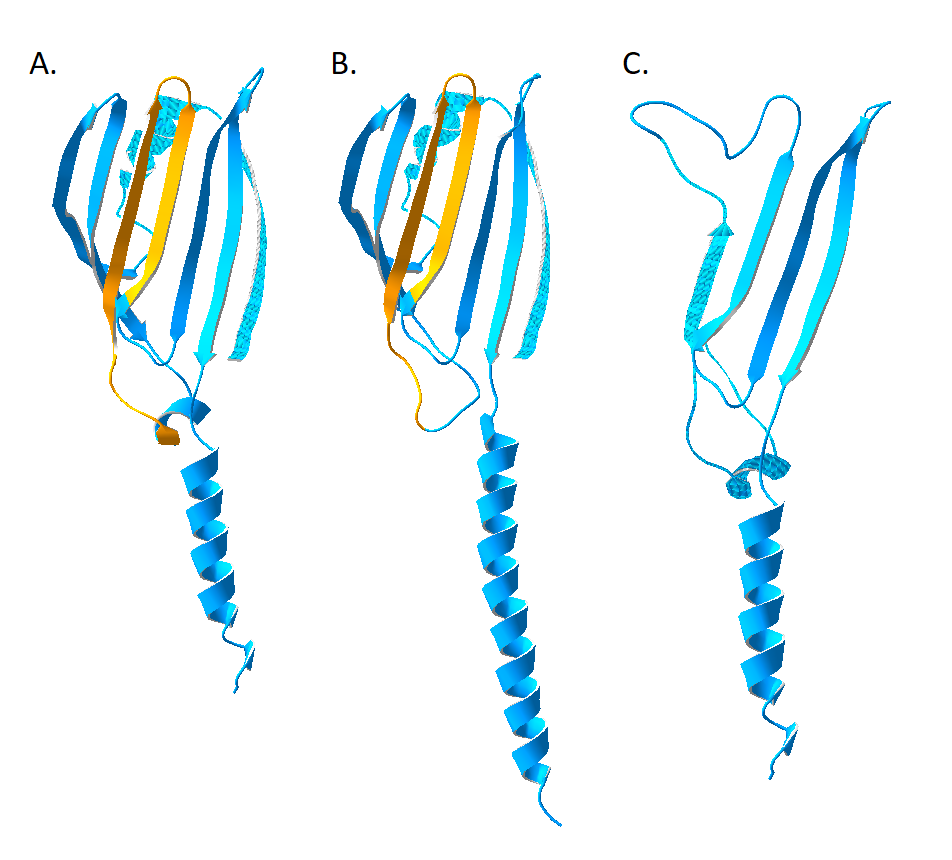

Supplement: S2 Fig — A. ENSBTAT00000081878.1, B. ENSBTAT00000046981.3 and C. ENSBTAT00000084244.1. The exon 9 (located between the amino acids 141 and 170) was identified in the exon expression analysis for the meat quality index and is represented by the golden segment. The models were constructed using the SwissModel server [46–48]. (PNG) [file pone.0240895.s002.png]

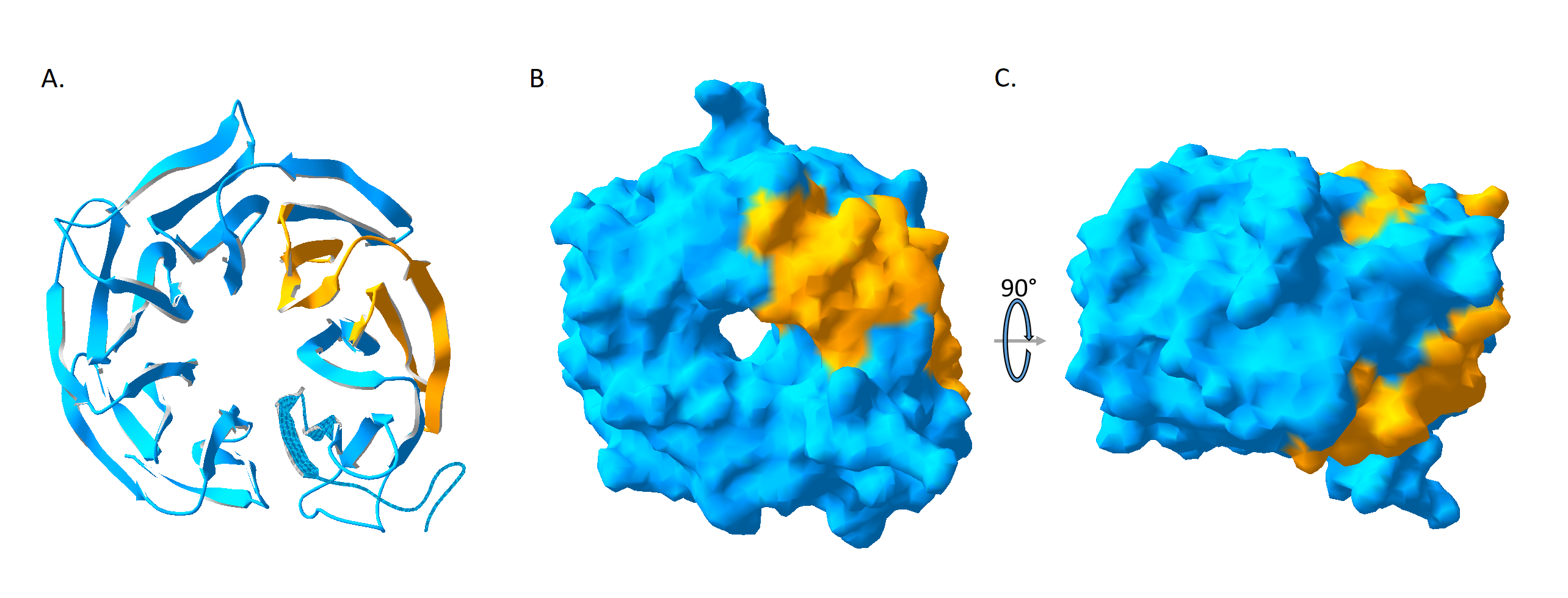

Supplement: S3 Fig — The exon 3 (located between the amino acids 99 and 148) was identified in the exon expression analysis for the meat quality index and is represented by the golden segment. B and C represent the ENSBTAT00000018753.4 isoform molecular surface and the golden region denotes the molecular surface of the exon 3. The models were constructed using the SwissModel server [46–48]. (PNG) [file pone.0240895.s003.png]

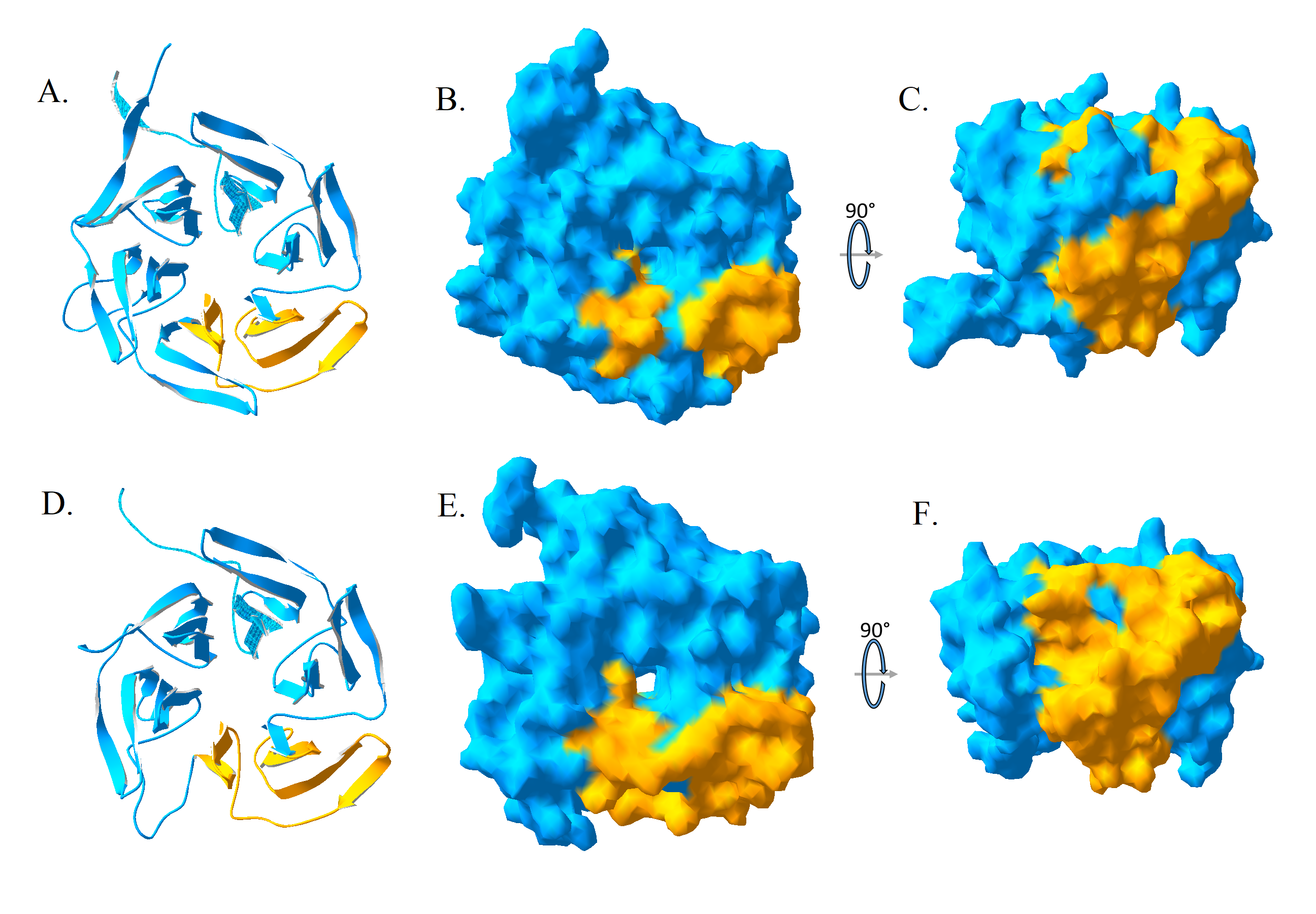

Supplement: S4 Fig — A. Ribbon representation of the analyzed KLHDC8B isoform ENSBTAT00000001298.3. The exon 3 (located between the amino acids 126 and 180) was identified in the exon expression analysis for the meat quality index and is represented by the golden segment. B and C represent the ENSBTAT00000001298.3 isoform molecular surface and the golden region denotes the molecular surface of the exon 3. D. Ribbon representation of the analyzed KLHDC8B isoform ENSBTAT00000001299.4. The exon 3 (located between the amino acids 126 and 180) is represented by the golden segment. E and F represent the ENSBTAT00000001299.4 isoform molecular surface and the golden region denotes the molecular surface of the exon 3. The models were constructed using the SwissModel server [46–48]. (PNG) [file pone.0240895.s004.png]

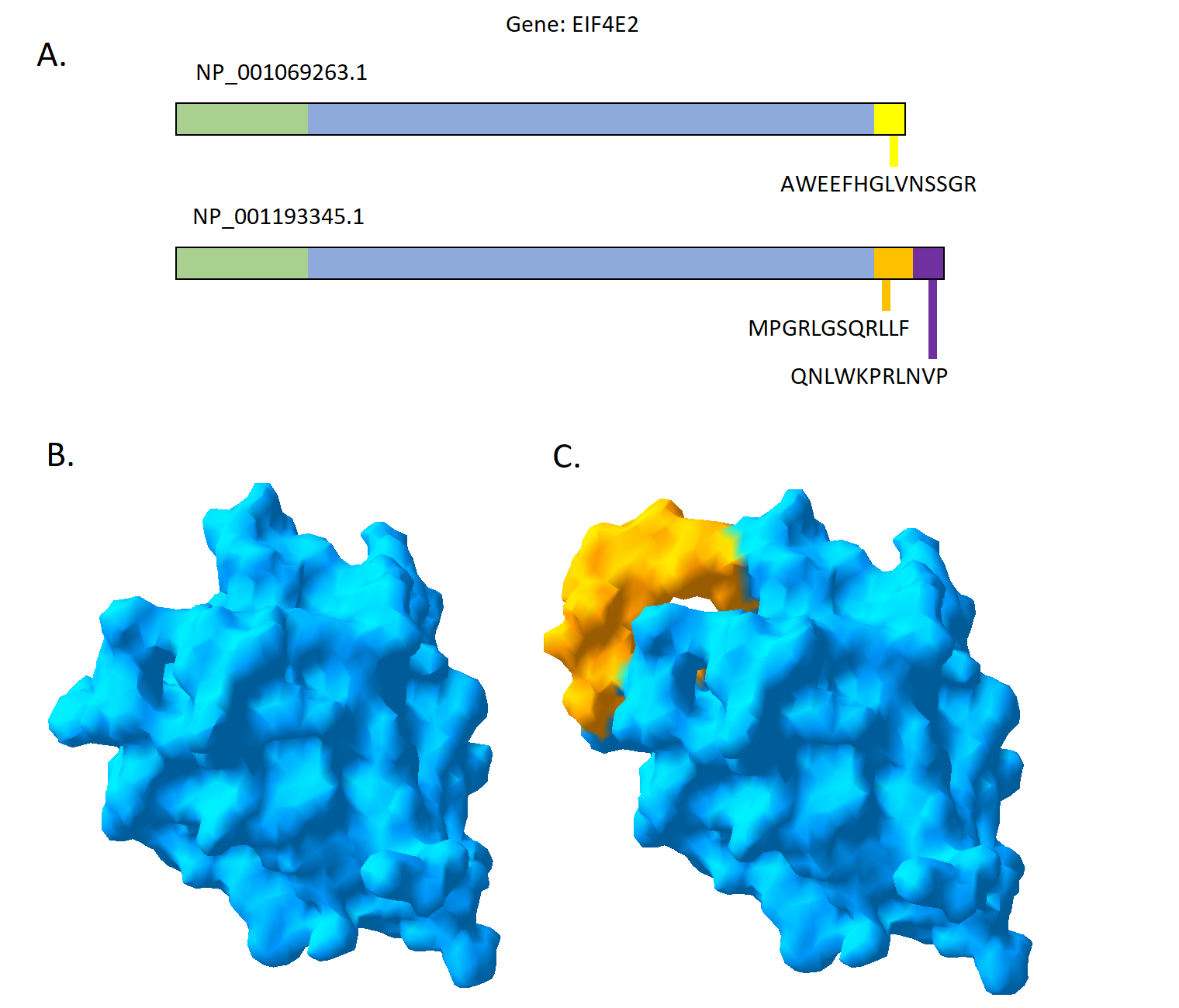

Supplement: S5 Fig — Blue and golden segments were modeled using the SwissModel server [46–48]. The molecular surface of the EIF4E2 isoforms NP_001069263.1 (B) and NP_001193345 (C) are presented and the golden region denotes the additional protein segment present in NP_001193345. The models were constructed using the SwissModel server [46–48]. (PNG) [file pone.0240895.s005.png]

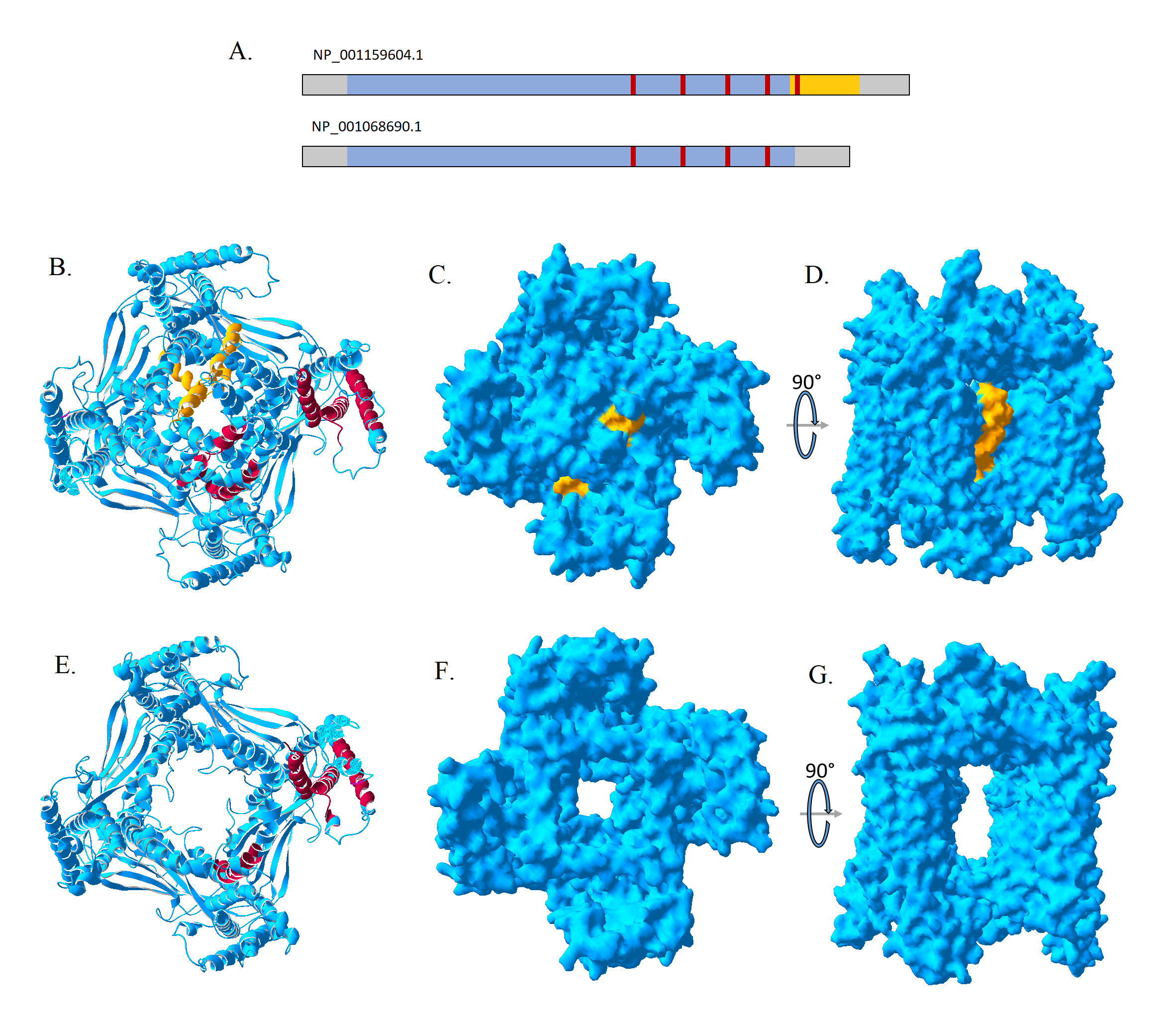

Supplement: S6 Fig — A. Comparison between the MCOLN1 isoforms NP_001159604.1 and NP_001068690.1. Blue, red and golden regions were modeled; red regions represent transmembrane segments. B. Ribbon representation of the analyzed MCOLN1 isoform NP_001159604.1. C and D represent the NP_001159604.1 isoform molecular surface and the golden region denotes the additional protein segment present in this isoform. E. Ribbon representation of the MCOLN1 isoform NP_001068690.1. F and G represent the NP_001068690.1 isoform molecular surface. (PNG) [file pone.0240895.s006.png]
